# Fertility Preservation: A Key Survivorship Issue for Young Women with Cancer

**DOI:** 10.3389/fonc.2016.00102

**Published:** 2016-04-25

**Authors:** Ana Milena Angarita, Cynae A. Johnson, Amanda Nickles Fader, Mindy S. Christianson

**Affiliations:** ^1^Department of Gynecology and Obstetrics, The Kelly Gynecologic Oncology Service, Johns Hopkins University School of Medicine, Baltimore, MD, USA; ^2^Department of Gynecology and Obstetrics, The Kelly Gynecologic Oncology Service, Johns Hopkins Hospital, Baltimore, MD, USA; ^3^Division of Reproductive Endocrinology, Department of Gynecology and Obstetrics, Johns Hopkins University School of Medicine, Baltimore, MD, USA

**Keywords:** fertility preservation, cancer survivorship, cryopreservation, fertility sparing surgery, counseling

## Abstract

Fertility preservation in the young cancer survivor is recognized as a key survivorship issue by the American Society of Clinical Oncology and the American Society of Reproductive Medicine. Thus, health-care providers should inform women about the effects of cancer therapy on fertility and should discuss the different fertility preservation options available. It is also recommended to refer women expeditiously to a fertility specialist in order to improve counseling. Women’s age, diagnosis, presence of male partner, time available, and preferences regarding use of donor sperm influence the selection of the appropriate fertility preservation option. Embryo and oocyte cryopreservation are the standard techniques used while ovarian tissue cryopreservation is new, yet promising. Despite the importance of fertility preservation for cancer survivors’ quality of life, there are still communication and financial barriers faced by women who wish to pursue fertility preservation.

## Introduction

**Figure 1:**
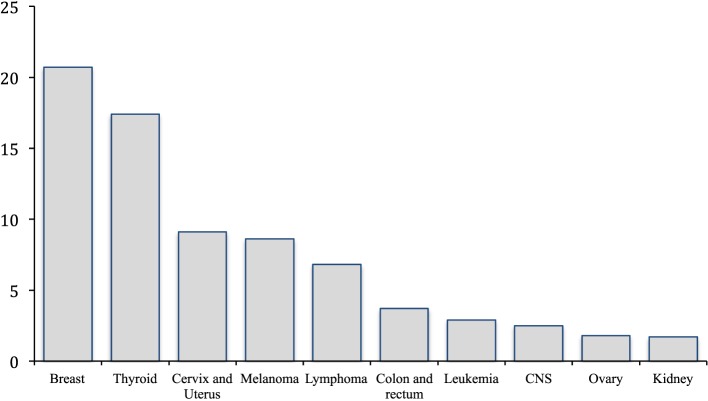
**Most common cancer in women 15–39 years old**. *Incidence rates per 100,000 (1).

Cancer in females of reproductive age accounts for nearly 10% of new cancer diagnoses, impacting 87 per 100,000 women annually (1). The most common cancers presenting in this cohort include breast, thyroid, cervical, uterine, melanoma, lymphoma, and colon cancer (Figure 1) (1). Over the past four decades, advances in surgery and adjuvant therapy have led to improved 5-year survival rates for breast (85.5%), endometrial (91%), cervical (83.2%), and ovarian cancers (79.5%) (2). These improved outcomes have resulted in an increased number of cancer survivors in the United States, rising from 3 million to nearly 14 million in the past 40 years (1). While improved treatments have increased survivorship rates in women with cancer, many therapies are harmful to the ovaries and put women at risk of premature ovarian failure and infertility. This is significant as nearly 25% of today’s cancer survivors are reproductive-aged woman who may wish to have children. With approximately half of women in the United States delaying childbearing into their thirties, the need for fertility preservation treatment has never been greater (3).

Fertility is a major concern for women with newly diagnosed cancer (4). A recent survey of young women undergoing treatment reported that 51.7% felt that having children was “most important” in their life (5). Potential fertility loss is related to emotional distress, fear, anxiety, and even moderate or severe depression. These symptoms, especially depression, are more commonly observed in young, non-white, and nulliparous women (6, 7). A number of studies suggest the risk of infertility with cancer therapy may adversely impact treatment decisions (8, 9). One survey evaluating women with early-stage breast cancer demonstrated that 0.6% of women elected not to receive chemotherapy due to fertility risks, whereas 1.9% chose one chemotherapy regimen over another to reduce impact on fertility. Similarly, 15.5% of women reported rejecting or shortening endocrine therapy for fertility reasons (9).

In recent years, an increasing number of female patients with cancer have presented to fertility specialists to discuss ways to preserve fertility prior to treatment to allow them to become biological mothers as cancer survivors (10). The most commonly utilized fertility preservation treatments include embryo, oocyte, and ovarian tissue cryopreservation (11). Other options include adoption or utilization of an egg donor, but studies show that the majority of women prefer to have biologically related children (10, 12, 13). The American Society of Clinical Oncology (ASCO) and the American Society of Reproductive Medicine (ASRM) both recognize fertility preservation as a key survivorship issue and recognize fertility preservation treatment as a key measure of quality of care (11, 14). Therefore, early referral to a fertility specialist and counseling women about their infertility risks prior to initiating cancer therapy are essential elements of comprehensive cancer care (15, 16). Although fertility preservation is a well-recognized survivorship issue, many barriers exist for women who may choose to pursue fertility preservation treatment (4, 17).

The objective of this review is to discuss the impact of cancer treatments on fertility in young, female cancer survivors and to appraise the fertility preservation treatment options available for reproductive-aged women. Additionally, we highlight research advancements in the field of fertility preservation and review current challenges faced by cancer survivors who may pursue fertility preservation treatment.

## Effect of Chemotherapy on Future Fertility

Females are born with approximately one million oocytes, stored as primordial follicles in the ovarian cortex. The number of follicles decreases with increasing age, eventually leading to cessation of menses and onset of menopause when their supply is depleted (18). However, in women who undergo cancer treatment, this process is often accelerated due to the cytotoxic effect of chemotherapeutic drugs. These agents primarily affect dividing cells and negatively impact follicular maturation. They are directly toxic to primordial follicles which may lead to premature ovarian failure (19). The risk of premature ovarian failure varies by age, chemotherapy agent or combination used, cumulative dose, and duration of treatment (18). Girls and young women have a rich ovarian reserve. When they undergo chemotherapy, they have a lower risk of ovarian failure than older women undergoing the same chemotherapy treatment (20). Larsen et al. demonstrated that in teenage females undergoing chemotherapy, the risk of premature ovarian failure increased by a factor of 4, while for women 21–25 years old the risk increased by a factor of 27 (21). Regarding the specific chemotherapy agent used (Table 1), alkylating agents deliver the highest risk of ovarian failure compared to other cytotoxic agents. Alkylators alter DNA base pairs, leading to cross-links and introducing single-strand DNA breaks (22).

**Table 1:** **Risk of ovarian failure according to the chemotherapeutic agent used**.

**High risk**
Alkylating agents
Cyclophosphamide
Ifosfamide
Nitrosoureas
Chlorambucil
Melphalan
Busulfan
Procarbazine
**Medium risk**
Alkylating agents
Cisplatin
Carboplatin
Doxorubicin
**Low or no risk**
Alkylating agents
Bleomycin (antibiotic)
Dactinomycin (antibiotic)
Antimetabolite agents
Methotrexate
Mercaptopurine
Fluorouracil
Antimicrotubule agents
Vincristine
Vinblastine

## Effect of Radiotherapy on Future Fertility

The oocyte is extremely sensitive to ionizing radiation, and radiation therapy causes a dose- and age-related reduction in the ovarian follicular pool (23). The extent of damage depends on volume treated, total radiation dose, fractionation technique, field arrangement, and patient age (24). Multiple fields are used to minimize radiation-induced toxicity by dividing the exposure of normal tissue into multiple different regions. Reproductive organs are affected directly, if they are included in the radiation field, or indirectly by scattered radiation (24). Current techniques such as intensity modulated radiation therapy (IMRT) and image guided radiation therapy (IGRT) have been used to maximize the dose to tumor and minimize toxicity to surrounding tissue (25).

As with chemotherapy, older females with fewer oocytes prior to treatment are most susceptible to ovarian failure (26). Additionally, radiation exposure to the uterus may produce tissue fibrosis, scarring, and decreased blood supply. This can result in infertility as well as poor obstetrical outcomes such as miscarriage and preterm birth (27–29). Moreover, brain irradiation can damage the hypothalamic–pituitary–gonadal axis, resulting in infertility due to anovulation secondary to hypothalamic amenorrhea (24).

## Effect of Surgery on Future Fertility

The diagnosis and initial treatment of gynecological malignancies implies performing surgical procedures to remove the affected reproductive organs. Both ovarian and endometrial cancers are surgically staged according to the International Federation of Gynecology and Obstetrics (FIGO), whereas cervical cancer is staged clinically (30, 31). The cornerstone of therapy for women with ovarian, fallopian tube, and peritoneal cancer is surgical cytoreduction to the presence of no gross residual disease, which is associated with increased survival (32). Ovarian cancer cytoreduction entails total hysterectomy, bilateral salpingo-oophorectomy, pelvic and para-aortic lymph node dissection, omentectomy, peritoneal biopsies, and collection of pelvic washings (32). Similarly, the standard staging procedure for endometrial cancer is total hysterectomy with bilateral salpingo-oophorectomy (30). Omentectomy is only performed when there is serous or clear cell histology. Moreover, pelvic- and para-aortic lymphadenopathy is performed selectively depending upon the presence of high-grade histology, extend of myometrial invasion, and tumor size >2 cm (33). Furthermore, one of the standard treatment options for women with early-stage cervical cancer (stage IA or IB1) is a hysterectomy (either simple or radical, depending on the clinical stage) with pelvic lymphadenectomy (34). All of the aforementioned surgical procedures have a direct impact on fertility rendering women with cancer infertile or menopausal after bilateral removal of the ovaries. Therefore, it is necessary to extensively counsel reproductive-aged women regarding the risks and benefits of surgical treatment and the implications of treatment on fertility. It is also vital to discuss more conservative surgical alternatives, if safe, which potentially are fertility-preserving.

## Fertility Preservation Options for Women Prior to Cancer Treatment

The ideal fertility preservation treatment should be individualized. It is dependent on the following patient factors: age, diagnosis, partner status, preference regarding use of donor sperm, time available before treatment, and her desire for future childbearing. The primary fertility preservation options today include embryo, oocyte, and ovarian tissue cryopreservation. Additional modalities include ovarian transposition and fertility-sparing surgery. Table 2 summarizes the fertility preservation treatment modalities detailed below.

**Table 2:** **Fertility preservation options for young cancer survivors**.

Fertility option	Ideal patient	Success rates	Benefits	Drawbacks
Embryo cryopreservation	• Has male partner or willing to use donor sperm • Has time for ovarian stimulation prior to treatment	• Cumulative pregnancy rate of 66% among women with cancer	• Standard technique • Predictable likelihood of success	• Financially costly • Requires time to stimulate ovaries to retrieve eggs
Oocyte cryopreservation	• Postpubertal women without a male partner or who do not wish to use donor sperm	• Pregnancy rate per cycle of 50.2% or per embryo-transfer 55.4%	• Standard technique • For women with ethical or religious objections to embryo • For women in countries where embryo cryopreservation is prohibited freezing • Greater reproductive flexibility	• Financially costly • Requires time to stimulate ovaries to retrieve eggs
Ovarian tissue cryopreservation	• Prepubertal girls or young women who do not have time for ovarian stimulation to retrieve eggs	• Pregnancy rate of 25% among women with cancer	• Experimental • No delay in the initiation of cancer therapy • Male partner and ovarian stimulation are not required	• Requires surgical procedure to harvest tissue • Ovarian tissue could potentially be seeded with malignant cells
Ovarian transposition	• Females with planned pelvic radiation therapy	• Success rate (preservation of short-term menstrual function) varies from 16 to 90%	• Ideal for patient requiring local pelvic radiation	• Requires surgical procedure
Fertility sparing surgery	• Women with certain early-stage gynecological malignancies	• Cumulative conception rate after trachelectomy 53% • Pregnancy rate after progestin therapy for endometrial cancer 34.8%	• Ovaries and/or uterus are preserved	

### Embryo Cryopreservation

Embryo cryopreservation is a widely established method for preserving reproductive capacity in women. Due to its high pregnancy rates, it is considered the “gold standard” fertility preservation option offering the best chances of a live birth in the future (18). Among women with cancer, one retrospective study reported a life birth rate of 44.4% (35).

Embryo cryopreservation requires a woman to undergo an *in vitro* fertilization (IVF) cycle that involves 10–14 days of controlled ovarian hyperstimulation utilizing gonadotropin injections. When follicles reach the appropriate size, oocyte retrieval is performed *via* transvaginal ultrasound-guided needle aspiration of follicular fluid while the patient is sedated. Oocytes are then fertilized *in vitro* and cryopreserved, typically at the blastocyst stage, for future use (18). Most women with a male partner choose embryo cryopreservation, whereas some women without a male partner may choose this method by using donor sperm (24). Studies have shown conflicting results regarding the number of eggs harvested from female cancer patients compared to those without cancer. In most studies, cancer survivors possess a lower, but still adequate, number of oocytes when compared to age-matched controls without cancer (36–39). Of note, however, Oktay et al. demonstrated that women with the *BRCA1* mutation appear to have a significantly lower ovarian response and produce fewer eggs per ovarian stimulation cycle (7.4 vs. 12.4) than women without the mutation (40).

The primary drawbacks to IVF include the time required, cost, and risk of ovarian hyperstimulation syndrome (OHSS) (6, 24). Medical expenses for an IVF cycle for fertility preservation are often not covered by private- or government-based insurance (41). The standard controlled ovarian hyperstimulation protocol starts at the onset of menses, which could result in a delay of 2–4 weeks (42, 43). While the conventional ovarian stimulation protocol is initiated at the beginning of the follicular phase, “random start” protocols may be initiated at the late follicular, periovulatory, or luteal phase (18). The latter protocols have similar numbers of oocytes retrieved, oocyte maturity, and fertilization rates than conventional-start protocols (44). Thus, random start protocols have proven to decrease total time to starting the IVF cycle, and cancer treatment, without compromising oocyte or embryo yield (44–47). An important risk to women undergoing IVF cycles is OHSS. Severe OHSS is a rare but serious complication of controlled ovarian stimulation. Women with OHSS may present with lower abdominal discomfort, nausea, vomiting, abdominal distension, ovarian enlargement, and ascites due to increased vascular permeability and third-spacing of fluid. Serious complications can include venous thromboembolism and stroke. Fortunately, there are techniques available to help prevent this iatrogenic condition, which may contribute to a delay in initiating cancer therapy (48).

### Oocyte Cryopreservation

Oocyte cryopreservation is a fertility preservation treatment most suitable for single or adolescent women. It is often chosen by women without partners or by those with a partner who desire maximum reproductive flexibility. Oocyte cryopreservation is also an option for women with religious or ethical objections to embryo freezing (14, 18). This fertility preservation modality was considered experimental until 2013. At that time, the Practice Committees of ASRM and the Society for Assisted Reproductive Technology (SART) concluded that mature oocyte cryopreservation should no longer be considered experimental. Therefore, they recommended this strategy for patients facing infertility due to chemotherapy or other gonadotoxic therapies when embryo cryopreservation is not possible (49).

Major drawbacks of oocyte cryopreservation include the time needed for ovarian stimulation as well as its decreased efficiency compared to embryo cryopreservation. Oocyte cryopreservation is technically more difficult than embryo cryopreservation due to the oocyte’s increased water content, making it more prone to cryoinjury. An egg’s meiotic spindle, cytoskeleton, and cortical granules are sensitive to damage by ice crystals during freezing and thawing (50). Also, hardening of the zona pellucida after ­cryopreservation hinders fertilization (51). However, in recent years, there have been remarkable advances in oocyte cryopreservation techniques, which have allowed 70–90% of cryopreserved oocytes to survive the freeze-thaw process (52, 53). Intracytoplasmic sperm injection (ICSI), in which a sperm is directly injected into a mature egg, allows fertilization despite zona pellucida hardening (54).

Slow-freezing and vitrification are the two primary cryopreservation techniques. Vitrification leads to an ultra-rapid freezing of cells or tissues by direct contact with liquid nitrogen without ice crystal formation. Vitrification has quickly evolved to become the most widely used method of egg cryopreservation due to the improved oocyte survival (85 vs. 65%) and fertilization rates (79 vs. 74%), compared to the slow-freeze method (55, 56). Conversely, the slow-freeze method involves use of a cryoprotectant that permeates and dehydrates the cell as it is slowly cooled, minimizing the intracellular ice crystal formation. After cryopreservation, when the woman is ready to pursue childbearing, the oocytes are thawed and fertilized *in vitro*. The patient can then undergo transcervical embryo transfer into the uterus, with excess embryos cryopreserved for future use.

*In vitro* fertilization outcomes with cryopreserved oocytes are comparable to fresh IVF and ICSI rates (57). One retrospective study showed that oocyte cryopreservation/thaw cycles had no significant difference in live-birth rate per mature oocyte retrieved when compared to fresh IVF cycles (2.7 vs. 4.2%, respectively) (58). Furthermore, randomized trials performed in infertile couples with supernumerary oocytes and donor oocyte populations also reported no significant differences in fertilization rate (88.3 vs. 84.9%) and clinical pregnancy rate per cycle (50.2 vs. 49.8%) between fresh and vitrified oocytes (59, 60).

### Special Considerations for Embryo or Oocyte Cryopreservation in Female Cancer Survivors

For women with estrogen-sensitive tumors (i.e., endometrial or estrogen receptor positive breast cancer), alternative ovarian stimulation protocols have been developed to circumvent the theoretical risk of supraphysiologic estradiol levels on cancer growth. Selective estrogen receptor modulators (tamoxifen) or aromatase inhibitors (letrozole) have been utilized for this purpose (42, 43). In such protocols, letrozole is used in addition to the standard gonadotropin dosing. Letrozole, most commonly used today, minimizes a women’s serum estradiol level during controlled ovarian hyperstimulation. Published reports demonstrate a similar number of total oocytes retrieved, length of ovarian stimulation, and fertilization rate when compared with protocols without letrozole (43, 44, 61). A prospective study of 79 women with breast cancer who underwent ovarian stimulation using letrozole plus gonadotropins or gonadotropins alone for oocyte/embryo cryopreservation demonstrated a recurrence rate and survival that was similar at 2- to 3-year follow-up to those who underwent no fertility-preserving procedure (62).

### Ovarian Tissue Cryopreservation

Ovarian tissue cryopreservation involves harvesting and freezing ovarian tissue, allowing preservation of oocytes within primordial follicles located in the ovarian cortex. In the future, the tissue can be autotransplanted into the cancer survivor or immature oocytes could be harvested and matured *in vitro* (18). Major benefits of ovarian tissue cryopreservation include that it can be performed in prepubertal females, it eliminates the need for sperm donation, and it can be performed immediately without a cancer treatment delay. Of note, it is the only fertility preservation option available for prepubertal girls. Moreover, tissue can be obtained quickly, and there is potential to have more oocytes available for future fertility treatment than can be retrieved from a single IVF stimulation (18, 63, 64). This procedure entails ovarian tissue harvesting prior to cryopreservation. Ovarian tissue is either harvested laparoscopically or at the time of a laparotomy under general anesthesia, regardless of menstrual cycle phase. Since many young girls undergo chemotherapy port placement under general anesthesia, laparoscopic ovarian tissue harvesting can be piggy-backed to this procedure. Due to the location of the oocyte-containing follicles in the outer millimeter of the ovary, cryopreservation can be limited to only a cortical strip of tissue. After cancer treatment, the ovarian cortex tissue is thawed and transplanted either orthotopically to remaining ovarian tissue or pelvic peritoneum, or it can be transplanted heterotopically to the forearm, abdominal wall, or chest wall (18, 65).

Ovarian cryopreservation should ideally be performed before the initiation of gonadotoxic therapy since certain chemotherapies can significantly decrease ovarian reserve with each cycle. A prospective study of women that underwent ovarian tissue cryopreservation compared the ovarian reserve of those who had received chemotherapy (ranged from one to seven cycles) with those who had not, with the aim of quantifying the effects of alkylating and non-alkylating agents on ovarian infrastructure. The authors demonstrated a significantly lower primordial follicle counts in women who received chemotherapy compared to controls. This effect was accentuated when women were treated with alkylating agents compared to those patients who did not receive these agents or did not receive chemotherapy (66).

Cryopreservation of ovarian tissue is a new, yet promising, fertility preservation treatment. The first live birth after ­autotransplantation of human ovarian tissue was reported in 2004 (67). To date, there have been at least 60 live births after ovarian tissue reimplantation (68). The slow-freezing cryopreservation technique was used in the majority of these cases while only two used vitrification. In a series of 80 cases from 4 fertility centers, the pregnancy rate was of 25.0%. Of note, two women each delivered three babies, reflecting potential long-term efficacy of ovarian cryopreservation (68, 69). Ideal candidates for this ­fertility preservation modality are girls/women under age 35 with at least a 50% risk of ovarian failure after cancer therapy (70).

### Ovarian Transposition

Ovarian transposition, or oophoropexy, is a strategy that can be offered to women with planned pelvic radiation. It is commonly considered for young women with locally advanced cervical cancer. This surgical procedure involves moving one (most commonly) or both ovaries out of the pelvis and away from the radiation field by laparoscopy or laparotomy (71). The ovary can be transposed to the lateral abdominal wall along the ipsilateral paracolic gutter, or with ligation to the uterosacral ligament for midpelvic or abdominal radiation, respectively (18). In all ovarian transposition cases, marking the boundaries of the ovary with surgical clips will help to identify the ovaries during radiotherapy mapping (18). This technique is commonly done unilaterally but a combined approach with cryopreservation of one ovary and transposition of the other can be also implemented (72). In the case of post-treatment failure of the non-transposed ovary, oocyte retrieval from the transposed ovary can be performed transabdominally if the ovary is not repositioned (18). The overall success rate as judged by preservation of short-term menstrual function is approximately 50%, although there is a wide variation in the reported success rates ranging from 16 to 90% (14, 73). Failure of this method is due to scatter radiation, compromise of the transposed ovary blood supply, patient age, radiation dose, whether the ovaries are shielded during the radiation procedure and whether concomitant chemotherapy is used (73). Complications related to ovarian transposition include infarction of the fallopian tubes and chronic pelvic pain (73, 74).

### Fertility-Sparing Surgery

Conservative surgical and medical techniques have been increasingly used for the management of early-stage gynecologic malignancies, given the impact of fertility preservation on quality of life (75). Fertility-sparing surgery entails the preservation of at least a portion of one ovary and the uterus, and it is more commonly offered to women with borderline ovarian tumors, non-epithelial ovarian cancers, early-stage cervical cancers, and select women with grade 1 endometrioid adenocarcinoma of the endometrium (75). Women with an apparent unilateral stage I borderline ovarian tumor or low grade ovarian malignancies who desire future fertility can be managed in some cases with a unilateral salpingo-oophorectomy, omental and peritoneal biopsies, and collection of pelvic washings rather than full staging for ovarian cancer (76). However, the National Comprehensive Cancer Network (NCCN) suggests consideration of completion surgery upon meeting childbearing goals for women with a remaining ovary (77).

There is limited data about the use of fertility-sparing surgery in women with early-stage epithelial ovarian cancer. In a large retrospective study of 240 women with epithelial ovarian cancer confined to the ovaries who underwent fertility-sparing surgery, 11.3% of the women relapsed and 4.6% died of progressive disease after a median follow-up of 9 years. The authors proposed a conservative approach (cystectomy or unilateral oophorectomy, omentectomy, pelvic washings, at least eight peritoneal biopsies, endometrial biopsy, and evaluation of pelvic and para-aortic lymph nodes) for appropriately selected young women with cancer. However, they recommended careful monitoring of women with grade 3 disease given the higher risk of distant recurrence (78).

Fertility-sparing surgery is particularly relevant for women with cervical cancer, given that this disease presents in women of reproductive age. Thus, women with tumors ≤2 cm and without evidence of obvious lymph node metastases can undergo cervical conization or radical trachelectomy, depending on disease stage, rather than radical hysterectomy (77). Conization is recommended for women with stage IA1 disease and without lymphovascular space invasion (79). A study of women 40 years or younger with stage IA1 disease using the Surveillance, Epidemiology, and End Results (SEER) database found no significant difference in 5-year survival between cervical conization and hysterectomy (80). Conversely, radical trachelectomy is recommended for women with stage IA1 disease with lymphovascular space invasion or stage IB1 disease (81). After the latter procedure, a 52.8% 5-year cumulative conception rate has been reported while reported preterm birth rate is in the range of 48–60% (81, 82). Several ongoing prospective trials, including a phase 2 trial at MD Anderson Cancer Center (NCT01048853), are underway to examine the safety of performing pelvic lymphadenectomy with cervical conization or simple hysterectomy for cervical cancer treatment. This study has an estimated enrollment of 100 participants and includes women with squamous cell carcinoma, FIGO stage IA2 or IB1, tumor diameter ≤2 cm, no lymphovascular space invasion on biopsy or cone and <10 mm of cervical stromal invasion (83).

In the case of uterine cancer, women with grade 1 or 2 endometrioid cancer confined to the endometrium may be candidates for progestin therapy such as megestrol acetate and deferral of surgical staging until after completion of childbearing. These women should have a dilation and curettage and imaging studies performed before medical therapy with the aim of excluding high-grade disease or advanced stages (84). A systematic review by Gunderson et al. of women treated with progestin therapy for grade 1 endometrioid carcinoma demonstrated a complete response rate of 48.2%. The time to complete response, which included women with hyperplasia, varied from 1 to 18 months (median 6 months). Moreover, the pregnancy rate for women with a history of carcinoma was 34.8% (84).

## Counseling and Referral of Women Interested in Fertility Preservation

It is well established that health-care providers should convey information about fertility risks and fertility preservation treatment to their patients as part of a comprehensive treatment plan. Open-ended dialog should include discussion of key points such as scientific data, advantages and disadvantages, anticipating delay of childbearing, patient preferences, and reproductive potential (11). Moreover, in order to improve information sharing, it is also beneficial to provide women with written material before and after counseling (16).

According to the ASCO Clinical Practice Guidelines, health-care providers should discuss with women interested in fertility preservation several key issues (11). The first key point is to discuss the feasibility of pursuing fertility preservation options depending on each patient’s recurrence risk and prognosis. Then providers should inform women of their individual risks of infertility or early menopause from oncologic therapy, taking into account individual factors. Patients should be told whether their treatment would place them in high, medium, low or non-existent risk. Next, fertility preservation treatment options, including those considered experimental, should be reviewed with their respective success rates. Health-care providers should communicate to women regarding the limited data available on oocyte cryopreservation and its decreased efficacy compared to embryo cryopreservation. It should be explained that these procedures may be subject to time constraints and treatment may be delayed. Patients should be informed that insurance coverage is improving for fertility preservation, and they should be encouraged to consult with their insurance companies. Providers should explain that even though there is a paucity of data, there appears to be no increased risk of cancer recurrence from fertility preservation interventions or pregnancy. Finally, an expeditious referral should be made to a fertility specialist for more information. Meeting with a social worker may also be beneficial for assessment of distress and to suggest advocacy organizations, which may provide financial resources.

## Barriers to Pursuing Fertility Preservation

Fertility preservation is of paramount importance for the quality of life of cancer survivors. Yet, this topic is not consistently addressed in clinical practice despite the aforementioned ASCO recommendations (14, 85). Moreover, there are still many factors that impact patients’ access to fertility preservation options. For example, both health staff and patients have their own concerns when it comes to discussing the effects that cancer therapy has on fertility. Although qualitative, a study reported that health-care providers voluntarily avoid this subject due to their beliefs that fertility in cancers such as Hodgkin’s lymphoma would not be affected by first-line chemotherapy and that fertility preservation treatments are not effective. Additionally, fertility preservation discussion may be avoided due to the sense of urgency in providing cancer care without delay (4).

Conversely, there are a number of reasons why young women may refrain from discussing the topic with health-care providers, such as being overwhelmed with their cancer diagnosis or unaware of the consequences that cancer treatment may have on their fertility. They often fear that delaying cancer treatment to pursue fertility preservation may negatively impact their survival (11, 86). These concerns reflect communication and information barriers, which can be addressed with education to both health-care providers and patients. Thus, it is important to inform patients that there is no significant delay in cancer treatment when pursuing fertility preservation options and that a prompt referral to a fertility specialist optimizes the lag time between diagnosis and start of cancer treatment (87, 88). A retrospective study demonstrated no difference in time from initial diagnosis to chemotherapy in women that underwent oocyte retrieval vs. women who did not (71 vs. 67 days, respectively, *p* < 0.27) (87). Likewise, another observational study of breast cancer patients showed that women referred to a subspecialist before surgery had a shorter time interval from initial diagnosis to initiation of ovarian stimulation (42.6 vs. 71.9 ± 30.7 days; *p* < 0.001, respectively) and to initiation of chemotherapy (83.9 vs. 107.8 days; *p* = 0.045) than women referred after surgery. Early referral can also allow repeated stimulation cycles, resulting in a larger number of oocytes or embryos for cryopreservation prior to cancer ­treatment (88).

Several studies have reported that up to 50% of young female cancer survivors did not receive sufficient education regarding fertility preservation options (89, 90). Furthermore, a population-based study demonstrated that only 56.3% of adolescent and young adults with cancer recalled discussing fertility preservation options and only 6.8% reported making arrangements to pursue any of those options. The authors also described that those discussions were less likely to occur if women were raising children or if they lacked private insurance. Additionally, 38% of the women reported not making arrangements for fertility preservation because they were unaware of the options, whereas 19% reported having cost issues. Strikingly, the study showed that men with cancer were more than twice as likely as women to report discussion of fertility preservation options and to make arrangements for fertility preservation (85). The sex differences found in these and other studies may be related to the costs and complexity of female fertility preservation options and to the fact that oocyte cryopreservation was experimental when women in the study were initially diagnosed (4, 85).

In addition to unmet communication needs, financial expenses are one of the most relevant barriers that cancer survivors face when making a decision about their reproductive future. The current costs of ovarian stimulation drugs ($2000–$5000), egg harvesting ($5000–$8000), annual storage ($500–$1000/year), and each attempt at embryo transfer ($4000–$5000) make it challenging to cover these expenses out-of-pocket (41). Unfortunately, insurance does not cover fertility preservation treatment for most female patients. The laws and regulations that address insurance coverage for fertility treatment define infertility as an inability to conceive after 1 year of regular and unprotected intercourse and do not mention the infertility caused by cancer therapy. Thus, there are no codified insurance mandates that would cover the expenses for fertility treatment specifically of cancer survivors (41). Moreover, as the laws pertaining to insurance coverage for infertility and IVF procedures vary among and within states, the obstacles that the survivors encounter when attempting to assess these services also vary widely (41). Conversely, due to the experimental nature of ovarian tissue cryopreservation, health insurers are not required to cover this service. This therefore limits the options for fertility preservation for prepubertal girls and young women (41).

Rationale behind the lack of insurance coverage for assisted reproductive technology in cancer patients are related to the view of these procedures as elective and not medically necessary (91). Fortunately, in recent years, there has been a slight increase in insurers covering fertility preservation treatment on a case-by-case basis. This highlights the importance of advising patients to contact their insurance companies regarding insurance coverage. Patients should also be encouraged to reach out to non-profit organizations that provide women with financial assistance for preservation treatment (92).

## Conclusion

Fertility preservation has become a significant aspect of comprehensive cancer care (24). The idea of not having a child of her own is a key source of distress in women with cancer undergoing gonadotoxic therapy. Health-care providers should discuss with women about their fertility wishes and counsel them regarding fertility preservation treatment options. Moreover, determining the need and best technique for fertility preservation requires an individualized assessment that is best performed by a fertility specialist. Barriers to fertility preservation counseling and receiving treatment continue to exist.

## Author Contributions

AA contributed to conception and design, manuscript writing, and final approval of the manuscript. CJ contributed to conception and design, manuscript writing, and final approval of the manuscript. AF contributed to conception and design, manuscript writing, and final approval of the manuscript. MC contributed to conception and design, manuscript writing, and final approval of the manuscript.

## Conflict of Interest Statement

The authors declare that the research was conducted in the absence of any commercial or financial relationships that could be construed as a potential conflict of interest.
